# Impact of national volume-based procurement policy on drugs treating chronic Myelogenous Leukemia

**DOI:** 10.1186/s41043-025-00829-y

**Published:** 2025-03-31

**Authors:** Zhao Yang, Yiran Li, Siyu Liu, Xiao Han, Yongyi Wu, Xiaohan Fan, Yuan Li, Bin Jiang

**Affiliations:** 1 Peking University First Hospital, Beijing, 100034 China; 2 Research Center of Public Policy, Peking University, Beijing, 100034 China; 3 School of Economics, University of Chinese Academy of Social Sciences, Beijing, 102844 China; 4 Shanxi Medical University, Jinzhong, 030001 Shanxi Province China; 5 School of Public Health, Shanghai Jiaotong University, Shanghai, 200025 China; 6 School of Public Health, Kunming Medical University, Kunming, 650500 Yunman Province China; 7 Scientific Research Department, Peking University First Hospital, Beijing, 100034 China; 8 Department of Hematology, Peking University First Hospital, Beijing, 100034 China; 9 Peking University School of Pharmaceutical Sciences, Beijing, 100871 China

**Keywords:** National volume-based procurement, Imatinib, Affordability, Chronic Myelogenous Leukemia

## Abstract

**Background:**

Tyrosine kinase inhibitors (TKIs) have been shown to improve survival rate in chronic myeloid leukemia (CML) patients, but their high costs impose a significant economic burden. This study aimed to evaluate the impact of China’s National Volume-Based Procurement (NVBP) policy on the affordability, procurement volume, and costs of these drugs.

**Methods:**

Based on the data of drug procurement transactions in the database of China Health Insurance Bureau, this study analyzed the impact of National Volume-Based Procurement policy on the affordability, Defined Daily Doses (DDDs), expenditure, and Defined Daily Dose cost (DDDc) of three tyrosine kinase inhibitor (Imatinib, Nilotinib, and Dasatinib) in 25 provinces and hospitals of different levels from January 2019 to December 2020 in China by using an interrupted time series model.

**Results:**

After the implementation of the procurement policy, the unit price of policy-related Imatinib decreased, leading to increased affordability and a rise of 146.9 thousand DDDs in the month of implementation (*p* < 0.05), while expenditure remained unchanged. The DDDs and expenditures for Nilotinib and Dasatinib did not show significant changes. The procurement volume of bid-winning generic Imatinib increased by 215.2 thousand DDDs in the month of policy implementation (*p* < 0.001), with usage share rising from approximately 40% to 80%. Conversely, the DDDs of non-bidding generic and originator drugs significantly decreased post-implementation. The DDDc of Imatinib reduced 69.5 in the month of NVBP implementation (*p* < 0.001). Furthermore, the DDDc of both bid-winning generic and non-bidding generic Imatinib significantly decreased in the month of policy implementation (*p* < 0.001).

**Conclusions:**

The National Volume-Based Procurement policy effectively reduced the unit price of relevant drugs and increased their utilization, thereby improving drug affordability and reducing the financial burden on CML patients requiring long-term treatment.

## Background

Chronic myeloid leukemia (CML) is a hematopoietic stem cell malignancy characterized by myeloproliferative neoplasms. The translocation of chromosomes 9 and 22 forms a new chromosome Ph. The protein *BCR::ABL* is the gene expression product of fusion gene *BCR::ABL* located on the chromosome Ph. With the tyrosine kinase activity, protein *BCR::ABL* promote proliferation and inhibit apoptosis of hematopoietic stem cells continuously, resulting in the disease development of over 95% patients [1, 2]. CML progresses through three distinct phases: the chronic phase (CP), the accelerated phase (AP), and the blastic phase (BP). Most patients are diagnosed in the CP, where the disease progresses slowly, is usually asymptomatic, and has the best prognosis. However, if left untreated, it can advance to more severe stages within 3–5 years [2, 3]. The annual incidence of CML is 1–2 per 100,000 worldwide, accounting for 15% to 20% of all adult leukemias [4]. In China, epidemiological surveys conducted in regions such as Inner Mongolia, Shanghai, and Jiangsu report an annual incidence rate of chronic myeloid leukemia (CML) ranging from 0.39 to 0.55 per 100,000 population, with a median age of onset between 45 and 50 years [5].

Tyrosine kinase inhibitors (TKIs) targeting the kinase expressed from the *BCR::ABL* gene have been recommended for first-choice treatment of CML in several guidelines [6, 7]. Imatinib, the first-generation TKI approved for CML, was launched in the United States in 2001 and has improved the 10-year survival rate of CML patients from 50 to 85–90%. However, 40–50% of patients did not respond to Imatinib treatment due to the development of resistance or adverse events [8]. This led to the development of second-generation TKIs. The objective response rates (ORRs) for first-generation and second-generation TKIs are 67.8% and 55.6%, respectively [9]. Currently, six *BCR::ABL* targeting TKIs are approved by the United States Food and Drug Administration for CML treatment: first-generation TKI Imatinib, second-generation TKIs Dasatinib, Nilotinib, and Bosutinib, and third-generation TKIs Ponatinib and Asciminib. The selection of TKIs in clinic is based on risk scores, toxicity profiles, patient’s age and ability to tolerate treatment, and the presence or absence of comorbidities. Meanwhile, TKIs for different targets continue to be developed [6]. Thus, for most patients, CML has become a chronic and manageable condition.

Although TKIs have significantly improved the survival rate of CML patients, their high cost remains a significant burden. Even Imatinib, with its patent expired and a generic version available in China (Table 1), imposes substantial financial strain. As of 2017, the monthly cost of Imatinib and Dasatinib in China was approximately US$3549 (11,200 mg) and US$693 (2800 mg), respectively. Adjusted for purchasing power parity, these prices were US$6661 and US$1301, while the per capita GDP of China in 2017 was just US$9,481.88 [10]. For CML patients requiring lifelong medication, the long-term financial burden is considerable, with 85% experiencing catastrophic medical expenditures after five years of TKI usage [11]. The high cost of treatment with anticancer drugs is a global issue, with studies indicating the worldwide cost of cancer treatment reached $196 billion in 2022, and 79% of anticancer drugs costing more than $100,000 annually over the past five years [12].

**Table 1:** Prices and monthly expenditures of TKIs

Drug	Manufacturer	Specifications	Unit price (US$)		Monthly expenditure (US$)	
			Pre	Post	Pre	Post
Imatinib	Chiatai	100mg	13.2	9.8	2380.3	1759.2
	Hansoh	100mg	15.8	10.4	2835.9	1869.0
Nilotinib	Novartis	Per mg	0.5	0.5	11,762.0	11,762.0
Dasatinib		Per mg	1.8	1.7	5457.0	5213.3

In China, the National Health Insurance Bureau (NHIB) has conducted National Volume-Based Procurement (NVBP) for generic medicines to reduce drug prices and improve affordability. The NVBP secures large orders for bid-winning pharmaceutical companies in exchange for lower prices, with hospitals prioritizing these drugs and ensuring timely payments to reduce transaction costs. The first round of NVBP was launched in March 2019 in 11 cities, expanding to a national level after December 2019. By December 2023, the ninth round of centralized national volume purchasing covered 374 medicines, achieving an average price reduction of over 50%. Imatinib was included in the 11 pilot cities in March 2019 and negotiated nationwide by the end of 2019.

This study aims to assess the impact of NVBP on TKIs for CML. We analyzed drug procurement data from January 2019 to December 2020 from over 20,000 hospitals across 25 provinces in China. Using the Interrupted Time Series (ITS) model, we evaluated the effects of this policy intervention on the procurement volume (DDDs), expenditures and DDDc of three TKIs. This study provides valuable insights into the effectiveness of NVBP and offers scientific evidence to support the further promotion of such policies to enhance medication affordability.

## Methods

### Data sources

This study extracted drug procurement data from China Health Insurance Bureau, covering 25 provinces from January 2019 to December 2020. The data included details such as generic name, drug manufacturer, formulation, package specification, unit price, procurement time, procurement volume, procurement province, and the name of the medical institution. We aggregated the procurement volume and expenditures by active ingredient to a monthly and national level to evaluate the overall impact of NVBP on the procurement volume and price of three TKIs.The average daily wage data at the provincial level is obtained from *China Statistical Yearbook-2020* and *China Statistical Yearbook-2021*.

### Drugs of interest

TKIs targeting at *BCR::ABL* tyrosine kinase protein for the treatment of CML, including Imatinib, Nilotinib, and Dasatinib, were included in the study according to the ATC/DDD Index 2024 [13]. Among these, only Imatinib was included in the NVBP, while Nilotinib and Dasatinib were not. Other relevant TKIs introduced in China after January 2019 were excluded from this study.

In this study, we categorized Imatinib into four subgroups according to manufacturers: bid-winning generic, non-bidding generic, non-bidding originative, and nonequivalent, to analyze the impact of NVBP on Imatinib’s sales structure. Pharmaceutical companies meeting the standards of drug efficacy equivalence evaluation were eligible to participate in the NVBP bidding. Companies that did not pass the equivalence evaluation were classified as Nonequivalent. Only the winning companies were guaranteed large purchase orders and required to sell the drug at a reduced price.

### Outcome variables

The aim of NVBP is to lower drug prices, making them more accessible and affordable for patients. Therefore, we examine the effects of such policies on drug unit prices and patient affordability (Eq. 1). Affordability is measured by the financial burden on patients, defined as the monthly expenditure on drugs per individual divided by the average daily wage in 2019. A ratio of less than 1 indicates that the drug is considered affordable [14].

1 $${\text{Provincial }}\;{\text{Affordability = }}\frac{{{\text{Monthly}}\;{\text{ Drug }}\;{\text{Expenditure}}}}{{{\text{Provincial}}\;{\text{ Daily }}\;{\text{Wage}}}}$$

Subsequently, we evaluate the impact on DDDs and total drug expenditures. NVBP aims to enhance patient access to drugs by reducing unit prices, which is expected to increase procurement volumes. DDDs, a metric introduced by the World Health Organization (WHO) to compare drug consumption, represent the total monthly prescription volumes divided by the Defined Daily Dose (DDD)[13]. Drug expenditures are denoted in million CNY.

Lastly, we assess the changes in Defined Daily Dose Cost (DDDc). This metric reflects the average daily cost borne by patients when using the drug. DDDc is calculated by dividing the total daily drug expenditures by DDDs. Consequently, DDDc reflects a combined outcome influenced by prescription volume and total drug expenses, providing a significant measure of the impact of strategic purchasing on daily patient drug costs.

### Statistical method

We used the interrupted time series (ITS) model to quantify the impact of NVBP on the DDDs, expenditures, and DDDc of drugs. The ITS model provides estimates of both the instantaneous impact on drug, and the change in the trend of drug prices and procurement volumes after the policy occurs. The ITS model shown in Eq. 2.

2 $$Y_{t} = \beta_{0} +\beta_{1}   \times Time_{t}  + \beta_{2}  \times Policy_{t} + \beta_{3}  \times Trend_{t} + \varepsilon_{t} $$

where ${Y}_{t}$ is the DDDs, expenditures, and DDDc of drugs at month $t$. ${\text{Time}}_{t}$ indicates number in months at time $t$ from the start of the observation period (January 2019); ${\text{Policy}}_{t}$ refers to the dummy variable indicating before and after intervention of the NVBP policy, where 0 represents before the policy intervention and 1 represents after the intervention.; ${\text{Trend}}_{t}$ equals zero until January 2020 and equals the number of months after January 2020 thereafter. ${\beta }_{0}$ is the constant term, represents the baseline level of the outcome variable at t = 0. ${\beta }_{1}$ represents the estimated trend of the independent variable with respect to the unit time variable t before the implementation of the NVBP policy.${\varepsilon }_{t}$ is the estimated value of the random error term.

The coefficients of interest are ${\beta }_{2}$ and ${\beta }_{3}$. ${\beta }_{2}$ estimated the instant change, and ${\beta }_{3}$ estimated the change in the growth trend of the DDDs, expenditures, and DDDc, after NVBP. The sum of ${\beta }_{1}$ and ${\beta }_{3}$ is the slope after the intervention is implemented.

We further calculated the estimated percent change as follows: we first calculated the estimate for the last period, that is, the sum of the estimate of ${\beta }_{0}$, the estimate of ${\beta }_{2}$, the estimate of 24 times ${\beta }_{1}$, and the estimate of 12 times ${\beta }_{3}$. Since ${\beta }_{1}$ depicts the estimated time trend (24 months) and ${\beta }_{3}$ estimates the change in trend after the policy is implemented (12 months), we multiplied these two coefficients by the corresponding number of months. Second, we computed a counterfactual estimate of the value of the last period assuming no strategic purchase, that is ${\beta }_{0}$ plus 24 times ${\beta }_{1}$. Finally, we used the estimated last period value divided by the estimated counterfactual last period value to get the estimated percent change.

The Durbin-Watson statistics was used to estimate residual autocorrelations. A p-value of less than 0.05 was considered statistically significant. All models were run using the statistical software Stata/SE 15.1, StataCorp.

This study did not involve patient or public participation and therefore did not require an ethical statement or patient informed consent.

## Results

### Impact of NVBP on unit price and affordability

Table 1 illustrates the changes in the prices of Imatinib, Nilotinib, and Dasatinib before and after the national implementation of the NVBP policy. Following the policy’s implementation, the monthly expenditure and unit price of Imatinib produced by Chiatai decreased by 26.1% and 25.8% ($3.4 per unit), respectively. Similarly, the monthly expenditure and unit price of Imatinib produced by Hansoh decreased by 34.1% and 34.2% ($5.4 per unit), respectively. As Nilotinib is under a national negotiation agreement (2018-12-01 to 2020-11-30), its price remains unchanged. The unit price (tablet) for Dasatinib decreased slightly after the implementation of the NVBP policy.

**Fig. 1:**
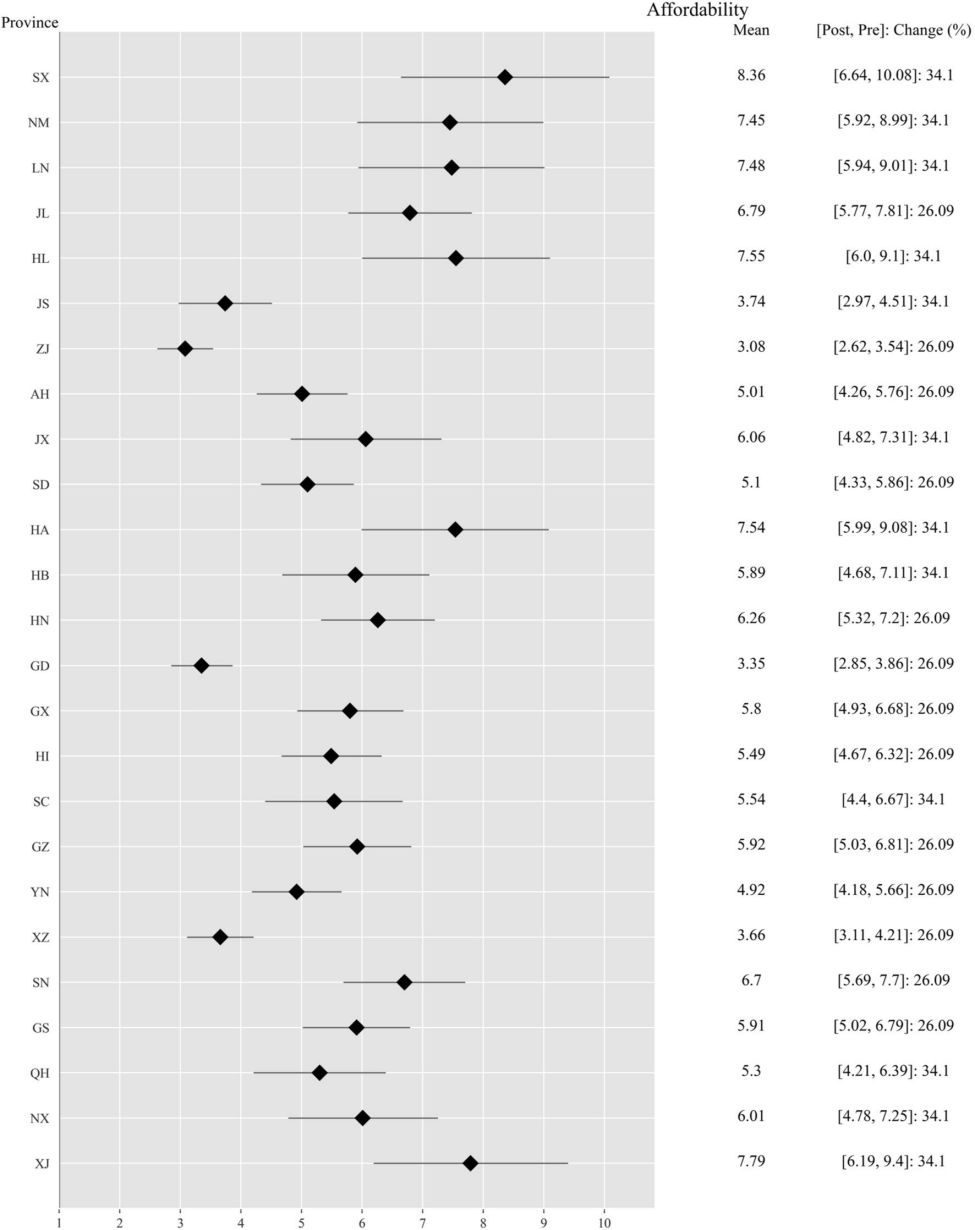
Forest plot of Imatinib

Figure 1 shows the changes in the affordability of Imatinib by province. Prior to the implementation of the NVBP policy, the affordability ranged from 3.5 to 10.1 with a mean of 6.9. After the NVBP policy was implemented, affordability of Imatinib improved from 2.6 to 6.6, representing a maximum decrease of 34.1%. The average affordability across all provinces after NVBP implementation was 4.8.

### Impact of NVBP on procurement volume

**Fig. 2:**
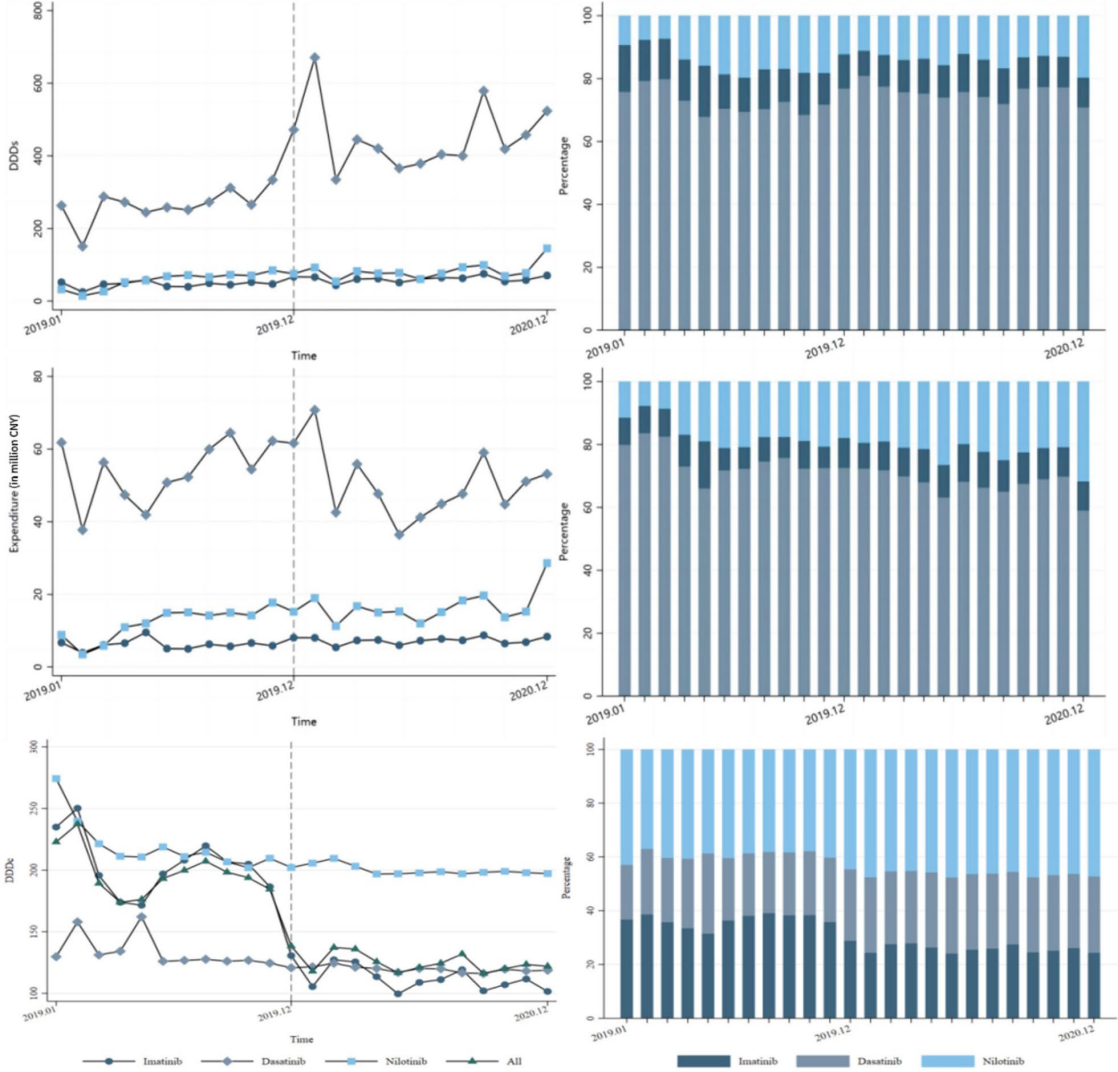
Procurement volume (DDDs), expenditure and DDDc of antileukemics

Figure 2 and Table 2 illustrate the changes observed in the three TKIs. Imatinib was the most frequently used TKI for CML treatment in 2019 and 2020. Following the national expansion of the NVBP in December 2019, there was a significant increase in its purchase volume. Specifically, after the NVBP implementation, the volume of Imatinib increased by 146.9 thousand DDDs in the month of policy implementation (*p* < 0.05).

**Table 2:** Procurement volume (DDDs), expenditure and DDDc of Imatinib, Dasatinib, and Nilotinib (ITS)

Tyrosine kinase inhibitors	Imatinib					Dasatinib	Nilotinib
Specific Categories	Bid-winning Generic	Non-bidding Generic	Non-bidding Originative	Nonequivalent	Whole		
Procurement volume (in thousand DDDs)							
Pre-Intervention Slope	1.261	4.081**	2.849***	−0.077	8.114	0.566	6.051**
	(6.008)	(1.382)	(0.715)	(0.270)	(7.300)	(0.835)	(1.611)
Change in Slope	2.731	−6.993***	−3.570***	−0.389	−8.220	−0.116	−3.393
	(7.610)	(1.751)	(0.906)	(0.342)	(9.247)	(1.058)	(2.041)
Change in Intercept	215.200***	−47.870***	−15.940*	−4.514	146.900*	9.412	−21.860
	(51.36)	(11.82)	(6.115)	(2.310)	(62.41)	(7.139)	(13.77)
Constant	98.88*	73.11***	33.40***	10.65***	216.00***	42.41***	19.57
	(40.75)	(9.374)	(4.851)	(1.833)	(49.51)	(5.664)	(10.93)
N	24	24	24	24	24	24	24
Adjusted R Squared	0.825	0.802	0.528	0.731	0.647	0.491	0.628
DW	2.584	1.762	1.633	2.061	2.333	2.424	1.785
P for DW	0.116	0.677	0.624	0.801	0.320	0.210	0.566
Expenditure (in million CNY)							
Pre-Intervention Slope	0.034	0.262*	0.881	−0.004	1.172	0.001	1.129**
	(0.330)	(0.0995)	(0.556)	(0.0183)	(0.848)	(0.117)	(0.330)
Change in Slope	0.149	−0.485***	−1.481*	−0.025	−1.843	0.031	−0.654
	(0.419)	(0.126)	(0.705)	(0.0232)	(1.074)	(0.149)	(0.418)
Change in Intercept	9.400**	−4.034***	−9.227	−0.357*	−4.218	0.966	−4.427
	(2.825)	(0.850)	(4.757)	(0.157)	(7.251)	(1.004)	(2.822)
Constant	8.12**	5.57***	32.12***	0.73***	46.54***	6.12***	5.24*
	(2.241)	(0.675)	(3.774)	(0.124)	(5.753)	(0.797)	(2.239)
N	24	24	24	24	24	24	24
Adjusted R Squared	0.746	0.853	0.483	0.749	0.154	0.221	0.559
DW	2.526	1.685	1.539	2.119	1.928	2.314	1.774
P for DW	0.146	0.522	0.549	0.703	0.838	0.382	0.605
DDDc							
Pre-Intervention Slope	−0.499**	−0.400	−25.780***	0.142	−2.383	−1.948*	−4.651***
	(0.165)	(0.221)	(3.530)	(0.262)	(1.647)	(0.790)	(0.941)
Change in Slope	0.392	−0.409	23.150***	−0.670	0.886	1.578	3.976**
	(0.209)	(0.280)	(4.471)	(0.332)	(2.086)	(1.001)	(1.192)
Change in Intercept	−21.380***	−9.456***	40.370	−11.620***	−69.500***	−2.002	7.993
	(1.413)	(1.892)	(30.17)	(2.241)	(14.08)	(6.757)	(8.047)
Constant	81.51***	75.78***	908.90***	68.45***	218.80***	145.60***	247.90***
	(1.121)	(1.501)	(23.94)	(1.778)	(11.17)	(5.361)	(6.384)
N	24	24	24	24	24	24	24
Adjusted R Squared	0.984	0.946	0.839	0.897	0.896	0.548	0.713
DW	2.385	1.321	0.727	1.516	1.195	2.701	0.959
P for DW	0.266	0.118	0.00158	0.244	0.0668	0.0386	0.167

Standard errors in parentheses; * *p* < .05, ** *p* < .01, *** *p* < .001

**Fig. 3:**
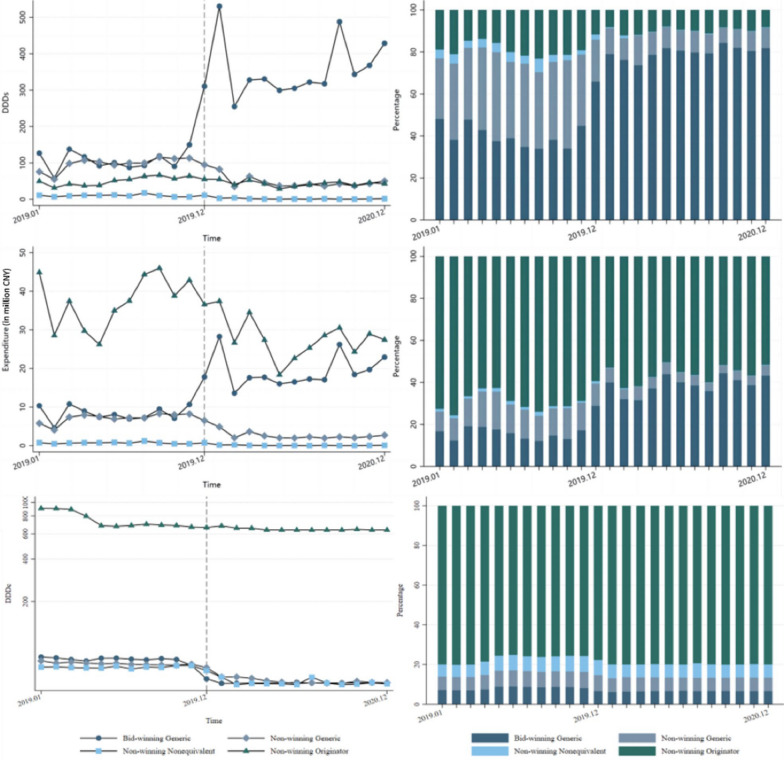
Procurement volume (DDDs), expenditure and DDDc of specific categories of Imatinib

Table 2 and Fig. 3 show the changes of the four subgroups of Imatinib. The four subgroups are bid-winning generic, non-bidding generic, non-bidding originative, and nonequivalent. The procurement volume of bid-winning generic drugs immediately increased by 215.2 thousand DDDs in the month NVBP implemented (*p* < 0.001). The trend of volume of non-bidding generic drugs and originative drugs shifted to downward after NVBP (*p* < 0.001), non-bidding generic drugs decreased by 47.87 thousand DDDs (*p* < 0.001) and non-bidding originative drugs dropped by15.94 thousand DDDs (*p* < 0.05).

### Impact of NVBP on expenditures

Figure 2 indicates that Imatinib holds the largest share of the market, with much higher utilization and expenditure compared to Nilotinib and Dasatinib. The price reduction effect of NVBP resulted in an increase in the procurement volume of Imatinib without a significant increase in its total expenditure. Instead, the expenditure on Imatinib fluctuated slightly below pre-NVBP levels. The expenditure of Dasatinib and Nilotinib, which were not included in the NVBP, were not significantly affected by the NVBP.

Despite the decrease in the unit price of bid-winning generic drugs, the procurement volume of Imatinib increased, leading to an expenditure rise of 9.4 million CNY in the month the policy was implemented (*p* < 0.01). In contrast, expenditures for non-bidding generic drugs declined, showing a downward trend after the NVBP took effect (*p* < 0.001) and decreased 4.034 million CNY in the policy implementation month (*p* < 0.001). Similarly, the expenditure on non-bidding originative drugs trended downward after the NVBP (*p* < 0.05), and expenditure of nonequivalent drugs dropped 0.357 million CNY in the implementation month (*p* < 0.05).

### Impact of NVBP on DDDc

The Defined Daily Dose Cost (DDDc) is the average cost of a drug per day, which is an important measure for assessing the daily drug burden on patients. Following the implementation of the NVBP policy, there was an immediate decrease in the Defined Daily Dose cost (DDDc) of Imatinib, which successfully bid, dropping by 69.5 CNY (*p* < 0.001).

For Imatinib, both bid-winning generic and non-bidding generic experienced immediate reductions in DDDc (Table 2), with reductions of 21.38 and 9.456 (*p* < 0.001) respectively. The DDDc of nonequivalent drugs also decreased in the month the NVBP was implemented (*p* < 0.001). While the original drug did not show a significant immediate change due to the policy, the trend in its DDDc was significantly impacted by the NVBP (*p* < 0.001).

### Heterogeneous policy effects across hospital types

**Fig. 4:**
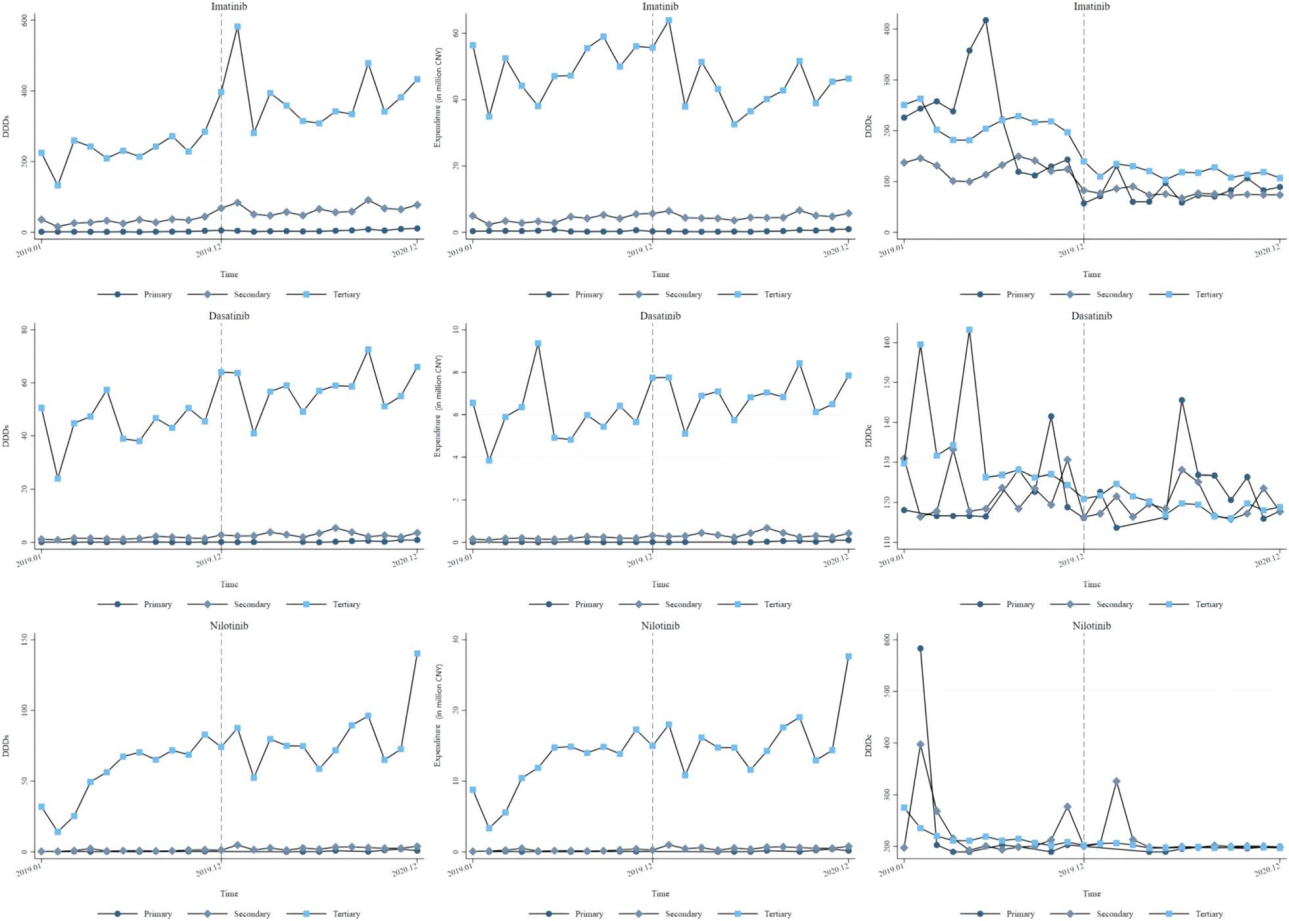
Procurement volume (DDDs), expenditure and DDDc stratified by level of hospitals

Table 3 and Fig. 4 display the effects of NVBP on different types of hospitals. In China, hospitals are categorized into three tiers based on the scope of medical services they provide, with tertiary hospitals primarily managing CML treatment. Most antileukemic drug use is concentrated in tertiary hospitals, but after the NVBP, the utilization of Imatinib increased in secondary hospitals.

**Table 3:** Procurement volume(DDDs), ependiture and DDDc stratified by level of hospitals (ITS)

Antileukemic	Dasatinib			Imatinib			Nilotinib		
Hospitals’ level	Primary	Secondary	Tertiary	Primary	Secondary	Tertiary	Primary	Secondary	Tertiary
Procurement Volume (in thousand DDDs)									
Pre-Intervention Slope	−0.000	0.068	0.496	0.164	1.370	6.580	0.012	0.027	5.981**
	(0.0168)	(0.0743)	(0.817)	(0.153)	(1.048)	(6.361)	(0.0411)	(0.0628)	(1.582)
Change in Slope	0.062**	−0.045	−0.134	0.364	−0.438	−8.286	0.154*	0.0357	−3.520
	(0.0209)	(0.0941)	(1.035)	(0.194)	(1.328)	(8.057)	(0.0617)	(0.0756)	(2.004)
Change in Intercept	−0.208	1.017	8.651	−1.128	19.940*	128.500*	−1.058	1.194*	−22.190
	(0.141)	(0.635)	(6.986)	(1.311)	(8.961)	(54.38)	(0.492)	(0.517)	(13.52)
Constant	0.085	1.09*	41.26***	1.10	23.24**	191.70***	0.086	0.76	18.96
	(0.119)	(0.504)	(5.543)	(1.040)	(7.109)	(43.14)	(0.286)	(0.431)	(10.73)
N	20	24	24	24	24	24	16	23	24
Adjusted R Squared	0.698	0.539	0.442	0.703	0.743	0.609	0.634	0.698	0.614
DW	1.765	1.638	2.432	1.218	1.949	2.456	2.008	2.751	1.756
P for DW	0.844	0.391	0.206	0.0692	0.939	0.198	0.172	0.0470	0.616
Expenditure (in million CNY)									
Pre-Intervention Slope	0.00005	0.008	−0.008	−0.004	0.178	0.998	0.001	0.013	1.114**
	(0.00194)	(0.00934)	(0.116)	(0.0168)	(0.0915)	(0.768)	(0.00811)	(0.0174)	(0.326)
Change in Slope	0.007**	−0.005	0.0284	0.058*	−0.162	−1.747	0.032*	−0.002	−0.673
	(0.00242)	(0.0118)	(0.147)	(0.0213)	(0.116)	(0.973)	(0.0122)	(0.0221)	(0.412)
Change in Intercept	−0.025	0.115	0.882	−0.359*	−0.040	−3.794	−0.208	0.199	−4.516
	(0.0164)	(0.0798)	(0.990)	(0.144)	(0.782)	(6.568)	(0.0971)	(0.149)	(2.783)
Constant	0.01	0.13*	5.97***	0.46***	2.91***	43.17***	0.025	0.13	5.09*
	(0.0138)	(0.0633)	(0.786)	(0.114)	(0.621)	(5.211)	(0.0565)	(0.118)	(2.208)
N	20	24	24	24	24	24	16	24	24
Adjusted R Squared	0.711	0.505	0.166	0.465	0.331	0.193	0.632	0.531	0.540
DW Before Adjustment	1.760	1.559	2.318	1.237	1.706	2.005	1.997	2.521	1.749
P for DW	0.840	0.288	0.383	0.0678	0.862	0.715	0.180	0.149	0.656
DDDc									
Pre-Intervention Slope	1.404	0.034	−2.704***	−23.460*	0.965	−2.464	−21.540	−5.256	−4.537***
	(0.895)	(0.503)	(0.519)	(10.07)	(2.286)	(1.797)	(10.30)	(4.693)	(0.947)
Change in Slope	−1.162	0.086	2.260**	25.540	−1.938	0.956	22.700	2.139	3.927**
	(1.115)	(0.638)	(0.625)	(12.49)	(2.843)	(2.277)	(15.47)	(5.944)	(1.199)
Change in Intercept	−7.502	−4.235	0.761	−68.470	−43.670**	−72.830***	42.420	26.680	7.181
	(7.546)	(4.303)	(4.270)	(56.50)	(11.83)	(15.37)	(123.3)	(40.12)	(8.093)
Constant	112.80***	122.50***	151.70***	389.80***	117.40***	229.70***	379.40***	263.70***	246.70***
	(6.352)	(3.414)	(3.568)	(79.47)	(18.50)	(12.19)	(71.71)	(31.83)	(6.421)
N	20	24	23	23	23	24	16	24	24
Adjusted R Squared	0.142	0.104	0.790	0.425	0.598	0.887	0.318	0.135	0.703
DW Before Adjustment	2.095	2.372	2.707	1.067	0.996	1.218	1.363	2.132	1.016
P for DW	0.502	0.193	0.0360	0.0354	0.0172	0.0844	0.798	0.593	0.272

Standard errors in parentheses; * *p* < .05, ** *p* < .01, *** *p* < .001

For procurement volume, Imatinib increased 19.94 thousand DDDs in secondary hospitals and 128.5 thousand DDDs in tertiary hospitals following the policy implementation (*p* < 0.05), whereas expenditures were not significantly changed. Before the NVBP, tertiary hospitals showed a growing trend in the utilization of Nilotinib (*p* < 0.01). In primary hospitals, the volume of Nilotinib had an increasing tendency (*p* < 0.05), and in secondary hospitals, procurement volume immediately rose by 1.194 thousand DDDs (*p* < 0.05) after the NVBP.

Regarding expenditure, Imatinib decreased by 0.359 million CNY at the time of policy implementation (*p* < 0.05). The expenditure of Nilotinib showed an increasing trend before the NVBP in tertiary hospitals (*p* < 0.01) and in primary hospitals (*p* < 0.05).

The DDDc of Imatinib instantly decreased 43.67 CNY in secondary hospitals (*p* < 0.01) and 72.83 CNY in tertiary hospitals after NVBP (*p* < 0.001). There was a slight increasing tendency in DDDc of Dasatinib in tertiary hospitals (*p* < 0.01), and a decreasing trend in DDDc of Nilotinib in primary hospitals (*p* < 0.01).

## Discussion

In this study, we used the Interrupted Time Series (ITS) model to analyze the impact of the National Volume-Based Procurement (NVBP) policy on the affordability, procurement volume, expenditure, and DDDc of three *BCR::ABL* inhibitors in China from January 2019 to December 2020. Our results show that the implementation of NVBP effectively reduced the price of these tyrosine kinase inhibitors (TKIs), increased their usage, improved affordability, and enhanced the well-being of chronic myeloid leukemia (CML) patients in China.

Despite the proven efficacy of TKIs for treating CML, high prices remain a significant barrier for many patients. For example, during 2015–2017, the average annual treatment costs for Imatinib, Dasatinib, and Nilotinib were $105,069, $116,729, and $112,780, respectively [15]. After the introduction of generic Imatinib in the United States in 2016, the price of the original drug decreased very slowly, and the generic was only 10% cheaper than the original in 2017. For CML patients who require lifelong medication, TKIs impose a substantial financial burden on both households and health insurance funds.

The NVBP policy promises large future order volumes to drug manufacturers in exchange for lower drug prices. Our research found that the unit price of Imatinib under NVBP decreased by 24.8% (Chia Tai-Tianqing) and 34.2% (Hansoh). Additionally, the average affordability across 25 provinces improved from 6.9 to 4.8. In contrast, the unit prices of similar TKIs did not change significantly during the same period. The quantitative estimation results from the ITS model confirmed these findings: while NVBP significantly increased Imatinib’s usage, it did not significantly raise total expenditure and further decreased the unit expenditure (DDDc). In previous studies, Jing Yuan et al. found that the volume-based procurement policy reduced the prices of 25 drugs by 21% to 96%, leading to an average drug cost reduction of 65.9% [16]. Therefore, it is believed that NVBP can reduce drug prices and improve the affordability of TKI drugs.

The impact of NVBP on drug prices and usage in China align with research findings from other countries. A study conducted in seven low- to middle-income countries showed that centralized procurement could reduce drug prices by at least 15% [17]. Similarly, Germany’s "Act to Reorganize the Market for Medicinal Products," implemented in 2011, led to a 24.5% decrease in incremental treatment costs of anticancer drugs compared to the previous nine years [18]. In Italy, price negotiations for innovative drugs between 2013 and 2017 resulted in an average price reduction of 32.2% [19].

In summary, our study, along with research on similar policies in other countries, shows that volume-based procurement is an effective method for controlling drug prices. This research can provide a valuable reference for other countries formulating policies to manage drug costs. For example, the Biden administration in the United States has initiated a drug price negotiation policy under the 2024 Medicare program to reduce procurement drug costs for Medicare patients. These policies could benefit from China’s experience with volume-based procurement.

Our study further reveals that the implementation of NVBP has not only dramatically increased the use of Imatinib but has also altered its market structure. Imatinib, a well-established drug for treating CML, already had a large market share before NVBP. Our results show a significant increase in the purchasing volume of bid-winning generic Imatinib following the policy’s introduction. This shift, where bid-winning drugs replace non-bidding ones, is due to the comprehensive implementation of strategies such as NVBP, consistency evaluation, Zero Markup Drug policy, and hospital performance assessments. These findings align with the policy recommendations of Ferrario et al. [20]. Thus NVBP might help lower the price of Imatinib overall. Although the utilization of Imatinib has increased, its expenditure has not increased significantly. This outcome can be attributed to reduced monthly costs and increased use of bid-winning generic Imatinib following NVBP. These findings are in line with the evaluation of NVBP’s early pilot program as studied by Chen et al. [21]. The introduction of the NVBP policy led to a significant decrease in DDDc of Imatinib, as well as notable reductions in both the winning and non-winning generic variants. This highlights how NVBP effectively lowered daily expenditures for patients.

We further conducted heterogeneity test across different levels of hospitals. Tertiary hospitals exhibited significantly higher procurement volumes and expenditures on TKI drugs compared to primary and secondary hospitals, consistent with prior studies by Fang et al. [22]. Given that tertiary hospitals in China generally offer lower health insurance reimbursements, patients with CML visiting these facilities often face higher financial burdens. Specifically, NVBP led to an increase in the procurement volume of Imatinib in secondary and tertiary hospitals while DDDc descreased in those hospitals. These changes underscore NVBP’s potential to alleviate financial strain on families and enhance healthcare accessibility.

Our study has several strengths. Firstly, our analysis is based on nationwide drug procurement data from all public healthcare institutions, enabling a thorough evaluation of NVBP’s impact on drug utilization across China. Secondly, China is the largest developing country in the whole world with a substantial population of low- and middle-income CML patients lends significant relevance to our analysis of TKI procurement. It also provides a reference for improving pharmaceutical policies in the future.

However, there are some limitations to our study. Firstly, it did not account for other factors that could influence policy effectiveness. Concurrent events, such as the Covid-19 epidemic, may have affected drug utilization, potentially biasing our results. Although Covid-19 primarily impacted Wuhan, Hubei Province during our study period, its timing overlapped with the policy intervention, which could have influenced the procurement and consumption of TKIs nationwide due to hospital operations and quarantine policies. Secondly, our study was based on drug purchasement data rather than drug utilization data. While procurement and utilization data generally align under most policies, discrepancies can occur. Despite these limitations, our study on NVBP demonstrated a notable reduction in the financial burden for patients with CML and a significant increase in the use of high-quality medications. These findings offer valuable insights for future implementations and refinements of similar policies.

## Conclusions

This study examined the impact of NVBP on TKIs used in treating CML. Our findings indicate that NVBP successfully reduced prices of bid-winning medicines, increased procurement volume, and encouraged substitution with generic drugs meeting efficacy equivalence standards. Given the substantial financial strain associated with CML treatment in China, NVBP holds promise for enhancing affordability of TKIs, thereby improving access to more cost-effective healthcare services for CML patients. Enhancing and expanding NVBP could significantly lower medication costs and enhance patients’ welfare. This study may serve as a useful reference for other countries considering similar policy implementations.

## Data Availability

The data that support the findings of this study are available from China Health Insurance Bureau, which were used under licence for the current study and so are not publicly available but are available from the corresponding author on reasonable request.

## Abbreviations

TKI: Tyrosine Kinase Inhibitors
CML: Chronic Myeloid Leukemia
NVBP: China’s national volume-based procurement
DDDs: Defined daily doses
DDDc: Defined daily dose cost
CP: Chronic phase
AP: Accelerated phase
BP: Blastic phase
NHIB: National health insurance bureau
ITS: Interrupted time series
WHO: World health organization
